# A worm's best friend: recruitment of neutrophils by *Wolbachia* confounds eosinophil degranulation against the filarial nematode *Onchocerca ochengi*

**DOI:** 10.1098/rspb.2010.2367

**Published:** 2010-12-22

**Authors:** Rowena D. E. Hansen, Alexander J. Trees, Germanus S. Bah, Udo Hetzel, Coralie Martin, Odile Bain, Vincent N. Tanya, Benjamin L. Makepeace

**Affiliations:** 1 Liverpool School of Tropical Medicine, School of Veterinary Science and Institute of Infection and Global Health, University of Liverpool, Liverpool L69 7ZJ, UK; 2 Institut de Recherche Agricole pour le Développement, Regional Centre of Wakwa, Ngaoundéré, BP 65 Adamawa Region, Cameroon; 3 USM 307 Parasitologie Comparée et Modèles Expérimentaux, Muséum National d'Histoire Naturelle, 61 Rue Buffon, 75231 Paris Cedex 05, France

**Keywords:** onchocerciasis, eosinophilia, filariasis, innate immunity, interleukin-8, GRO

## Abstract

*Onchocerca ochengi*, a filarial parasite of cattle, represents the closest relative of the human pathogen, *Onchocerca volvulus*. Both species harbour *Wolbachia* endosymbionts and are remarkable in that adult female worms remain viable but sessile for many years while surrounded by host cells and antibodies. The basis of the symbiosis between filariae and *Wolbachia* is thought to be metabolic, although a role for *Wolbachia* in immune evasion has received little attention. Neutrophils are attracted to *Wolbachia*, but following antibiotic chemotherapy they are replaced by eosinophils that degranulate on the worm cuticle. However, it is unclear whether the eosinophils are involved in parasite killing or if they are attracted secondarily to dying worms. In this study, cattle infected with *Onchocerca ochengi* received adulticidal regimens of oxytetracycline or melarsomine. In contrast to oxytetracycline, melarsomine did not directly affect *Wolbachia* viability. Eosinophil degranulation increased significantly only in the oxytetracycline group; whereas nodular gene expression of bovine neutrophilic chemokines was lowest in this group. Moreover, intense eosinophil degranulation was initially associated with worm vitality, not degeneration. Taken together, these data offer strong support for the hypothesis that *Wolbachia* confers longevity on *O. ochengi* through a defensive mutualism, which diverts a potentially lethal effector cell response.

## 1. Introduction

The maternally transmitted α-proteobacterium, *Wolbachia*, infects an estimated two-thirds of all arthropod species [1], but it also has a more limited distribution in certain parasitic nematodes (the family Onchocercidae, superfamily Filarioidea, order Spirurida [2,3]; and the family Pratylenchidae, order Tylenchida [4]). In arthropod hosts, it expresses a diverse array of phenotypes from reproductive alterations including cytoplasmic incompatibility, male killing and feminisation [5], to mutualistic roles such as metabolic provisioning and protection against pathogens [6–8].

Filarial nematodes harbouring *Wolbachia* cause two major neglected tropical diseases, lymphatic filariasis (elephantiasis) and onchocerciasis (River Blindness), which are responsible for a combined global morbidity of greater than 6 million disability-adjusted life-years [9]. The obligate dependency of *Wolbachia*-positive filariae on their symbiont was demonstrated by prolonged antibiotic treatment of filarial infections in animal models, which led to stunting and sterilization [10] or death of adult worms [11]. These findings have been successfully translated into a treatment for individual patients suffering from filarial diseases [12]. Additionally, *Wolbachia* contributes to the clinical presentation of filarial infections, since bacterial products released from both living [13] and dead [14] worms activate the mammalian innate immune system, triggering the release of pro-inflammatory mediators.

The complete genome sequence of the *Wolbachia* strain (*w*Bm) from the human lymphatic filariid *Brugia malayi* revealed that the bacterium carries the genes required to synthesize riboflavin and haem, while its filarial host does not [15]. These data reinforced the prevailing perspective, initiated by the antibiotic chemotherapy experiments, that *Wolbachia* in filariae are ‘obligate mutualists’, in contrast with their close cousins in arthropods, which could be broadly described as ‘reproductive parasites’ [16].

In filarial nematodes, the precise mechanism by which *Wolbachia* depletion leads to adult worm death has not been elucidated. In *Onchocerca ochengi*, a parasite of cattle that represents the closest relative of the human pathogen *Onchocerca volvulus* [17], prolonged, intermittent antibiotic regimens are adulticidal [11,18]; whereas short continuous treatment is not [19]. Moreover, antibiotic chemotherapy radically alters the nature of the inflammatory response around the adult worms [20]. These parasites reside in fibrous nodules (onchocercomata) and can live for a decade, surrounded by a host infiltrate that consists primarily of neutrophils, which are stimulated by *Wolbachia*-derived molecules [21,22]. Following endobacterial clearance by oxytetracycline, the neutrophil population gradually recedes and is replaced by an influx of eosinophils [20], the classic effector cell in helminth infections, which are conspicuous by their scarcity in untreated onchocercomata. Thus, it is possible that the neutrophilic response is not simply an inevitable consequence of the presence of an endobacterium within a parasite of mammals, but instead a sublime example of defensive mutualism, in which the fitness of the worm is increased by an admixture of immunological signals. We tested this hypothesis by comparing the local immune response to adult *O. ochengi* treated with either an antibiotic (oxytetracycline) or a conventional adulticide (melarsomine), since if the observed eosinophilia is merely a non-specific response to dead or moribund worms, it should also occur following melarsomine chemotherapy.

## 2. Material and methods

### (a) Animals, field site and chemotherapy

Ngaoundéré Gudali cattle (*Bos indicus*) naturally infected with *O. ochengi* (greater than or equal to 30 palpable intradermal nodules per animal) were purchased from markets across the Adamawa Region of Cameroon and assembled at the Institut de Recherche Agricole pour le Développement (IRAD), Regional Centre of Wakwa.

The position of 23 nodules per animal was marked by peripheral tattoo and recorded by digital video and on a hard-copy ‘hide map’. Using a statistical calculator to generate random numbers, 15 cows were allocated to three groups of five through elimination by ear tag number, and one bull was assigned to each group (since sufficient female animals were not available). One group received oxytetracycline (OXY; Terramycin LA, Pfizer) at 10 mg kg^−1^ intravenously, daily for 14 days followed by 20 mg kg^−1^ intramuscularly, monthly for five months; a regimen that kills greater than 80 per cent of adult worms by 12 months after the start of treatment [19]. One group was treated with melarsomine (MEL; Cymelarsan, Merial) solution at 4 mg kg^−1^ intravenously, every other day for 3 days, which has been demonstrated to be 100 per cent adulticidal [23]. This drug kills the majority of worms by 20 weeks, although some nodules containing fragments of degenerated parasites may persist for several years after treatment [18]. The third group of animals served as untreated controls (CON). Nodules were excised under local anaesthesia at nine pre-determined time-points, and the sequence of nodulectomies was randomized (as for group allocation) with regard to their position on the animal. At every time-point, one nodule per animal was fixed by injection with 10 per cent neutral-buffered formalin, and another was preserved by injection with RNA*later* (Sigma) and stored at −20°C. A third nodule was removed at every other time-point and fixed by injection with 5 per cent glutaraldehyde in 75 mM sodium cacodylate buffer, pH 7.4 and stored at 4°C.

### (b) Histopathology of the bovine cellular response

Paraffin-embedded, formalin-fixed nodules were cut into 4 µm sections. For quantitative histopathological analysis (performed by an individual ‘blinded’ to treatment group), the sections were stained with Giemsa and digital images of the entire nodule at 20× magnification were obtained on an Eclipse 80i microscope (Nikon UK, Kingston-upon-Thames) using a DS-U1 camera control unit and NIS-Elements Basic Research 3.0 software. The diameter and area of each nodule were calculated; and granulocytes within a continuous transect of the nodule midsection were differentiated by eye at 200× [20], digitally tagged as neutrophils or eosinophils, and quantified within an overlaid grid. Counts were normalized to cells per square-millimetre. Degranulating eosinophils (DE) were semi-quantified on a four-point scale as number per worm section (0 = 0, 1 = <1, 2 = 1–10, 3 = >10) within each transect.

### (c) Spatial distribution of eosinophil degranulation in relation to worm integrity

In all nodules from 8, 12 and 24 weeks post-treatment (wpt) containing DE, the spatial distribution of eosinophil clusters containing greater than 10 cells was analysed in relation to the presence, proximity and integrity of worm sections by light microscopy. Three concentric zones around each eosinophil cluster were defined as: 0 = <50 µm; 1 = >50, <100 µm; and 2 = > 100, <150 µm. The structural integrity and reproductive status of the worm sections were scored according to a simplified version of the form proposed by Striebel [24], using categories presented in table 1.

**Table 1.** Associations between presence and integrity of *O. ochengi* adult worm sections in bovine nodules and degranulating eosinophil (DE) score at eight weeks post-treatment. (Bold type indicates statistical significance. The associations between degranulation and worm integrity were positive in all cases; i.e. DE scores were moderate and/or high where the sections exhibited normal structure. Sample sizes varied between analyses because only worm sections that could be scored unambiguously for each characteristic were included. Zone boundaries were measured in micrometres.)

observation	scoring system	value of *χ*^2^ or *r*_s_	*n*	association with DE score, *p*
presence of worm	absent = 0; present = 1	9.319^a^	135	**0.020**
proximity to worm (if present)	zone 0: <50; zone 1: >50, <100; zone 2: >100, <150	−0.334^b^	120	**<0.001**
genital tract integrity	damaged wall = 0; smooth wall = 1 (normal)	14.745^a^	43	**<0.001**
cuticle integrity	broken = 0; rough = 1; smooth = 2 (normal)	0.228^b^	115	**0.014**
hypodermal integrity	vacuolated = 0; thin = 1 (normal)	9.993^a^	111	**0.011**
reproductive status	non-productive = 0; productive = 1 (normal)	7.301^a^	109	**0.026**
intrauterine microfilariae	none = 0; present, damaged = 1; present, normal = 2	0.089^b^	113	0.350

^a^Fisher's exact test.

^b^Spearman's *ρ*.

*Wolbachia* were localized in worm sections by immunohistochemistry using a polyclonal antibody against *Wolbachia* surface protein (kindly donated by M. Casiraghi, University of Milan), as previously described [19]. Glutaraldehyde-fixed nodules were prepared for electron microscopy as detailed previously [19], and micrographs were obtained on an H-7100 transmission electron microscope (Hitachi High Technologies, Krefeld, Germany) fitted with a charge-coupled device camera (Hamamatsu, Massy, France) and a digital image acquisition software (Advanced Microscopy Techniques, Danvers, MA, USA). Bovine neutrophils and eosinophils were differentiated according to ultrastructural characteristics (see the electronic supplementary material, appendix S1, for details).

### (d) Analysis of gene expression following drug treatment *in vitro* and *in vivo*

*Aedes albopictus* mosquito cells naturally infected with *Wolbachia* (cell-line Aa23) were cultured in 25 cm^2^ flasks as previously described [25]. The cells were treated in quadruplicate for 3 or 7 days without treatment, or with melarsomine dihydrochloride (generously donated by C. Marcato, Merial, Toulouse) or doxycycline hyclate (Fluka) at three concentrations. The highest concentration applied (0.25 µg ml^−1^) represented the minimal bactericidal concentration for doxycycline against *Wolbachia* [26]. The viability of *Wolbachia* and host cell physiology was determined by quantitative reverse-transcriptase (qRT)-PCR, using assays for *Wolbachia* 16S rRNA and *A. albopictus* 18S rRNA as detailed elsewhere [25].

For gene expression analysis in nodules preserved in RNA*later*, the tissue was cut into pieces of 1–2 mm^3^ and homogenized at IRAD in Lysis-Binding Solution from an RNAqueous-Midi Kit (Ambion), using a T10 basic Ultra-Turrax disperser (IKA, Staufen, Germany) with an 8 mm dispersing element. The homogenates were stored at −80°C and shipped to the UK on dry ice, where the remainder of the kit manufacturer's protocol was followed. Total RNA was further purified by lithium chloride precipitation, dissolved in 0.1 mM ethylene diamine tetra-acetic acid, and quantified on a Nanodrop-1000 spectrophotometer (Thermo Fisher Scientific, Wilmington, DE, USA). If necessary, the RNA was concentrated in a Nanosep Omega 30K centrifugal device (Pall Life Sciences); and 1 µg was reverse-transcribed and assayed for *Wolbachia*, *O. ochengi*, and bovine transcripts using SYBR Green I (SensiMix Two-Step Kit, Quantace) on a MiniOpticon Real-Time PCR Detection System (Bio-Rad, Hemel Hempstead). For further details of qRT-PCR assay design, see the electronic supplementary material, appendix S2.

### (e) Statistical analysis

Two cows (one each in the CON and MEL groups) died from causes unrelated to this study before the experiment was completed, and data from these animals were excluded from the statistical analyses. After normalization by log transformation, independent gene expression data were analysed using univariate general linear model (GLM), whereas equivalent data from related samples were subjected to GLM repeated measures. The Friedman test (with exact significance) was applied to time-course data in the histopathological analyses. Since onchocercomata resolve after worm death, nodules could not be obtained from all pre-determined sites at later time-points. Therefore, data from 24 to 36 wpt, and 48 to 54 wpt, were collapsed into one category each in the Friedman analysis, incorporating mean values where more than one sample per animal was available. To test for associations or correlations between DE score and worm status, Fisher's exact test (for two categories) or Spearman's *ρ* (for three categories) was used, respectively. All analyses were performed in PASW Statistics 17.0 (SPSS Inc., Chicago, IL, USA), applying a critical probability of *p* < 0.05. For full details of GLM design and post hoc analyses, see the electronic supplementary material, appendix S3.

## 3. Results

### (a) Effect of melarsomine on *Wolbachia in vitro*

The results from treatment of the Aa23 cell-line demonstrated that conversely to doxycycline, melarsomine preferentially targets eukaryotic cells and it exhibits no inhibitory or bactericidal activity against *Wolbachia* (see the electronic supplementary material, appendix S4 for details).

### (b) Effects of treatments on nodule histopathology

**Figure 1.**
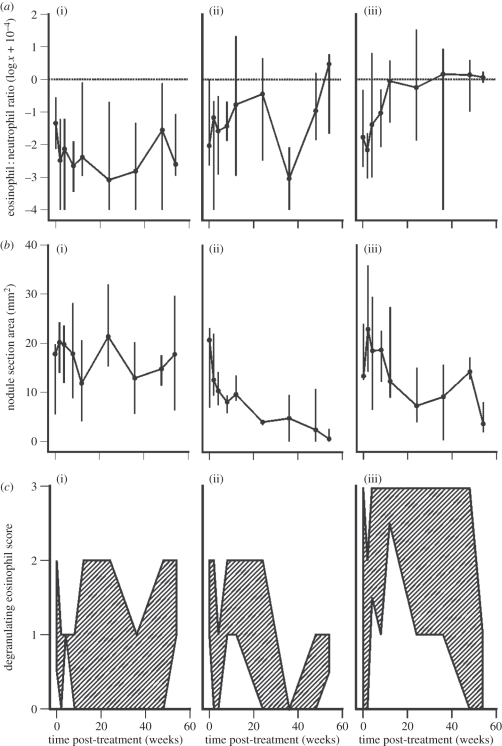
Bovine granulocyte responses in *O. ochengi* onchocercomata following (*a*(iii), *b*(iii), *c*(iii)) oxytetracycline (*n* = 6) or (*a*(ii), *b*(ii), *c*(ii)) melarsomine chemotherapy (*n* = 5), or (*a*(i), *b*(i), *c*(i)) in the absence of treatment (*n* = 5). (*a*) Eosinophil : neutrophil ratios (median and range). The horizontal dotted line represents parity between the two cell types. (*b*) Nodule section area (median and range). (*c*) Degranulating eosinophil scores per worm section (where 0 = 0, 1 = <1, 2 = 1–10, 3 = >10). The shaded area represents the region bounded by the median and maximum scores.

**Figure 2.**
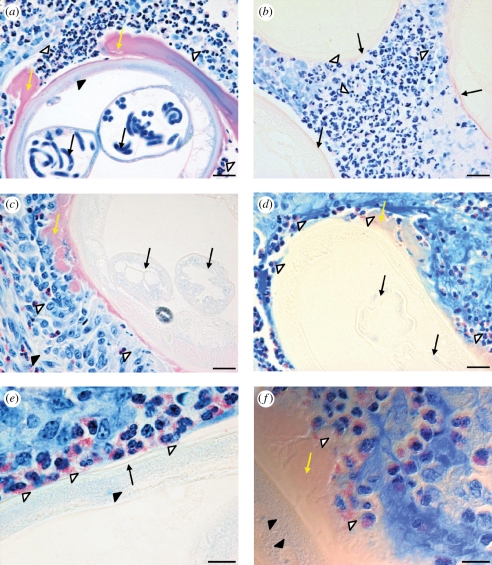
Histopathology of *O. ochengi* onchocercomata at 12 weeks. (*a*) Untreated onchocercoma with typical accumulations of neutrophils (open arrowheads) and Splendore-Hoeppli (SH) deposits (yellow arrows) on the worm cuticle. A cross section of an adult female worm shows the hypodermis (closed arrowhead) and microfilariae (black arrows) in the paired uteri. (*b*) A nodule from the melarsomine (MEL) group, displaying abundant neutrophils (open arrowheads) between the cuticles of three worm sections (black arrows). (*c*) A MEL-treated nodule with eosinophils (open arrowheads) and degranulating eosinophils (DE; closed arrowhead) some distance from a worm section. The worm has accumulated SH deposits (yellow arrow) and the uteri are empty (black arrows). (*d*) A nodule from the oxytetracycline (OXY) group shows numerous DE clusters (open arrowheads) around the cuticle, SH phenomenon (yellow arrow) and empty uteri (black arrows). (*e*) A higher power image from an OXY-treated nodule reveals DE and free granules (open arrowheads) in contact with the worm cuticle (black arrow) overlaying the hypodermis (closed arrowhead). (*f*) Phase-contrast image of a section from an OXY-treated nodule displaying eosinophil granules (open arrowheads) embedded in SH deposits (yellow arrow); filarial nuclei are also visible in the hypodermis (closed arrowheads). All sections were stained with Giemsa. Scale bars: (*a*—*d*) 20 µm; (*e*,*f*) 10 µm.

Quantitative analysis revealed striking differences in the nodular eosinophil : neutrophil ratio between treatment groups (figure 1*a*). In the CON group, neutrophils vastly outnumbered eosinophils (overall median ratio = 0.004) and there was no significant change in the composition of the granulocytic infiltrate over time (Friedman test: *χ*^2^ = 5.6, *p* = 0.507). A modest increase in the eosinophil : neutrophil ratio was observed in the MEL group, culminating in a shift towards eosinophilia in resolving nodules at the termination of the experiment, although this was not statistically significant (figure 1*a*; Friedman test: *χ*^2^ = 6.0, *p* = 0.473). However, there was a much larger and sustained expansion of the nodular eosinophil population in the OXY group, which peaked in a statistically significant, 275-fold increase in the median eosinophil : neutrophil ratio by 36 wpt (figure 1*a*; Friedman test: *χ*^2^ = 19.3, *p* < 0.001). Median nodule area gradually reduced during both adulticidal treatments (figure 2*b*) as the worms were eliminated and this was statistically significant in the MEL group (Friedman test: *χ*^2^ = 11.1, *p* = 0.012), although only a non-significant trend was apparent in the OXY group (Friedman test: *χ*^2^ = 10.3, *p* = 0.096). When DE adjacent to worm sections were analysed separately on a semi-quantitative scale, a clear distinction between the MEL and OXY treatment groups was observed (figure 1*c*). In the MEL group (in common with the CON group), the median score did not exceed 1 throughout the experiment, and no significant change over time was detected (Friedman test, MEL: *χ*^2^ = 4.9, *p* = 0.605; CON: *χ*^2^ = 3.9, *p* = 0.755). By contrast, there was a marked, statistically significant peak in DE in the OXY group at 12 wpt (figure 1*c*; Friedman test: *χ*^2^ = 11.9, *p* = 0.042) and the maximum score of 3 (greater than 10 DE per worm section) was only recorded herein.

The spatial distribution of granulocytic infiltration was investigated during the period of maximum divergence in DE activity between treatment groups (12 wpt). Onchocercomata from CON animals exhibited neutrophilic infiltrates in close proximity to the cuticle of the adult worms (figure 2*a*), whereas eosinophils were extremely scarce and located near the outer capsule. Extensive Splendore-Hoeppli phenomena were common around female worms (figure 2*a*). In the MEL group, neutrophils remained abundant between worm sections (figure 2*b*); although a modest increase in eosinophils was observed near to some female worms (figure 2*c*). However, eosinophils were very rarely in contact with the worm cuticle in the MEL group and degranulation was infrequent, usually occurring distally to worm sections (figure 2*c*). In marked contrast, eosinophils accumulated in layers around the worm cuticle in the OXY group, while neutrophils had become scarce (figure 2*d*). Furthermore, the majority of eosinophils surrounding the worms in the OXY group were undergoing degranulation (figure 2*d*). At higher magnification, clusters of eosinophil granules could be observed in direct contact with the epicuticle (figure 2*e*) or embedded within the Splendore-Hoeppli deposits adjacent to the cuticle (figure 2*f*).

### (c) Analysis of factors associated with eosinophil degranulation

To investigate the factors that may trigger eosinophil degranulation, nodules that exhibited DE in the transect analysis at 8, 12 or 24 wpt were carefully inspected to locate all clusters of greater than 10 eosinophils *in toto*. One-hundred-and-forty such clusters were identified, 89 per cent of which were in OXY-treated onchocercomata. At 8 wpt, the DE score for these clusters showed a weak but statistically significant negative correlation with neutrophil counts (Spearman's *ρ*: *r*_s_ = −0.259, *n* = 135, *p* = 0.002). At the same time-point, there was a statistically significant association (Fisher's exact test) between the DE score and the presence of worm sections in the microscopic field, and a significant positive correlation (Spearman's *ρ*) between the DE score and the proximity of worm sections (table 1). Moreover, there were statistically significant positive associations or correlations between the DE score and normal morphological features in the worm sections (smooth genital tract and cuticle, thin hypodermal cords and productive gonads), although not with intrauterine microfilariae *per se* (table 1). At 12 and 24 wpt, there were no significant associations between the DE score and these measures of morphological integrity. This was probably because less than 10 per cent of worm sections had a normal appearance at these time-points, producing extremely low expected frequencies in the analyses.

### (d) Effects of treatments on worm ultrastructure

**Figure 3.**
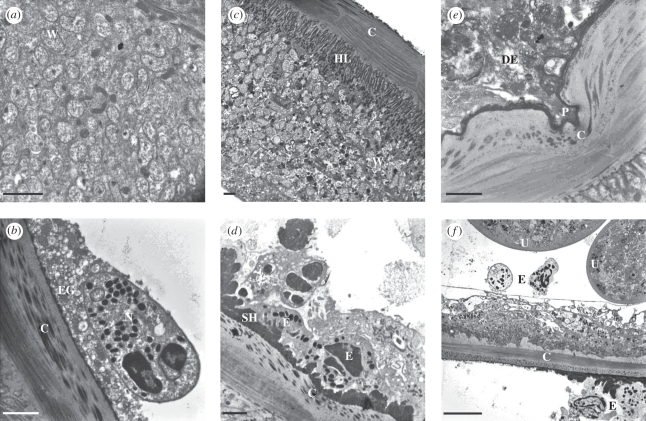
Ultrastructure of *O. ochengi* onchocercomata at 12 weeks. (*a*) Hypodermis of an untreated worm showing abundant *Wolbachia* endosymbionts (W). (*b*) The cuticle (C) of an untreated worm displaying normal structure, alongside an adherent neutrophil (N) surrounded by numerous extracellular granules (EG). (*c*) A worm section from the melarsomine (MEL) group displaying intact *Wolbachia* (W) beneath the hypodermal lamellae (HL) and the cuticle (C). (*d*) Eosinophils (E) attached to Splendore-Hoeppli deposits (SH) on the cuticle (C) of a worm from the MEL group. (*e*) The pseudopodium (P) of a degranulating eosinophil (DE) entering a cleft in the cuticle (C) of an oxytetracycline (OXY)-treated worm. (*f*) Two eosinophils (E) adjacent to the paired uteri (U) within the pseudocoelomic cavity of a worm from the OXY group; eosinophils are also visible on the host side of the cuticular (C) interface. Note the highly vacuolated hypodermis. Scale bars: (*a*–*e*) 2 µm; (*f*)10 µm.

In worm sections from the CON group at 12 wpt, *Wolbachia* endobacteria were extremely abundant in the hypodermis (figure 3*a*). Neutrophils adjacent to the cuticle were frequently observed undergoing degranulation in the CON group, although this did not cause any discernible damage (figure 3*b*). In the MEL group, *Wolbachia* remained at high densities in the hypodermal cords (figure 3*c*) in worms that were not in the advanced stages of resorption. Eosinophils were occasionally seen close to the cuticle, but degranulation was very infrequent and the outer layers of the cuticle remained unmodified (figure 3*d*). By contrast, the ultrastructure of nodules in the OXY group at 12 wpt revealed unique alterations. Eosinophil degranulation in direct contact with the worm cuticle was common and associated with pronounced deformation of the outer cuticular layers (figure 3*e*). Furthermore, parasite integrity was clearly compromised, as eosinophils could be observed free within the body cavity of the worms (figure 3*f*).

### (e) Effects of treatments on gene expression in onchocercomata

To determine the relative effect of MEL or OXY chemotherapy on the viability of *O. ochengi* and *Wolbachia* between 8 and 24 wpt, RNA transcripts of glutathione *S*-transferase 1a (*Oo*GST1a) were quantified from the former and 16S rRNA from the latter, and the ratio was calculated. Since nodules vary in worm content, the ratio between the expression levels of two genes is likely to be more informative than the absolute counts for either transcript alone. Both treatments showed a non-significant trend towards reduction in expression of *Oo*GST1a over this time period, which approached the critical probability only in the MEL group (GLM repeated measures, Tamhane's T2 post hoc test, MEL versus CON: mean difference = −2.3 log, *p* = 0.069; see the electronic supplementary material, appendix S5, for data and additional details of the analysis). However, for 16S rRNA, both treatments induced a significant decrease in transcript counts relative to the CON group, although this was more substantial in the OXY group (see the electronic supplementary material, appendix S5; GLM repeated measures, Tukey's post hoc test, MEL versus CON: mean difference = −2.1 log, *p* = 0.016; OXY versus CON: mean difference = −3.1 log, *p* = 0.001). Importantly, the 16S rRNA : *Oo*GST1a ratio was significantly reduced relative to the CON group only in OXY-treated nodules (see the electronic supplementary material, appendix S5; Tamhane's T2 test: mean difference = −2.1 log, *p* < 0.001); and furthermore, this ratio was significantly lower in the OXY group than in the MEL group (Tamhane's T2 test: mean difference = −2.4 log, *p* < 0.001). These data were corroborated by immunohistochemistry for *Wolbachia* surface protein in worm sections from 12 wpt, which showed retention of *Wolbachia* in worms that had not yet disintegrated after the MEL treatment; whereas staining in worms from the OXY group was weak or negative (see the electronic supplementary material, appendix S6).

**Figure 4.**
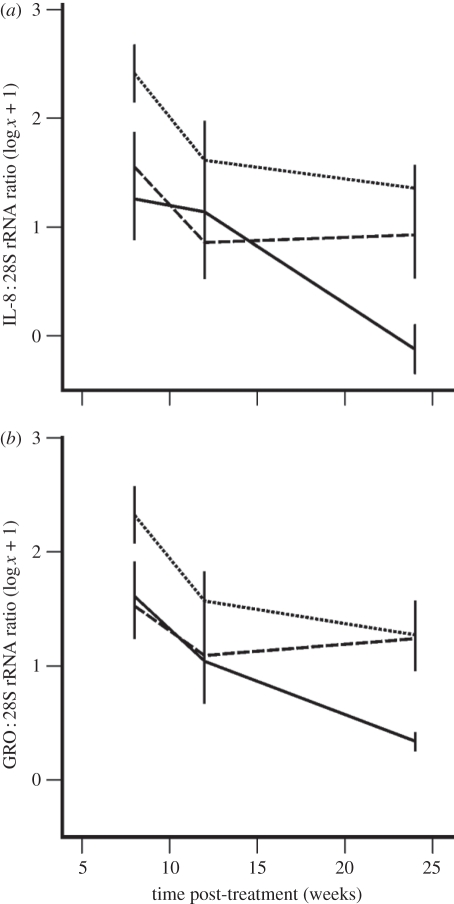
Quantification of bovine transcripts (normalized against 28S rRNA) for the neutrophilic chemokines (*a*) interleukin-8 (IL-8) and (*b*) the growth-regulated (GRO) protein family in onchocercomata treated with oxytetracycline (*n* = 6) or melarsomine (*n* = 5), or in the absence of treatment (*n* = 5). Lines represent the mean ± s.e. (*a*,*b*) Dotted line, control; dashed line, melarsomine; solid line, oxytetracycline.

To assess local cellular immune responses to different treatments in the bovine host, transcripts for key cytokines involved in the activation and chemotaxis of neutrophils (interleukin-8 (IL-8) and growth-regulated (GRO) family) and eosinophils (IL-5 and eotaxin) were quantified between 8 and 24 wpt. In the OXY group, the IL-8 : 28S rRNA ratio was significantly lower than that observed in the CON group (figure 4*a*; GLM repeated measures, Tukey's post hoc test: mean difference = −1.0 log, *p* = 0.010; see the electronic supplementary material, appendix S4, for additional statistical details); whereas in the MEL group, IL-8 levels were not significantly different to those in CON nodules (Tukey's test: mean difference = −0.7 log, *p* = 0.107). Similarly, the GRO : 28S rRNA ratio was significantly reduced in the OXY group relative to CON levels (figure 4*b*; GLM repeated measures, Tukey's post hoc test: mean difference = −0.7 log, *p* = 0.044), but not between the MEL and CON groups (Tukey's post hoc test: mean difference = −0.4 log, *p* = 0.296). However, neither treatment had a significant effect on the expression of IL-5 (GLM repeated measures, Tukey's post hoc test, MEL versus CON: mean difference = 0.09 log, *p* = 0.918; OXY versus CON: mean difference = −0.03 log, *p* = 0.991; data not shown) or eotaxin genes (GLM repeated measures, Tukey's post hoc test, MEL versus CON: mean difference = −0.5 log, *p* = 0.641; OXY versus CON: mean difference = −0.3 log, *p* = 0.814; data not shown).

## 4. Discussion

A role for the immune system in the mechanism of action of antibiotics against adult *Onchocerca* spp. was first identified in a sequential study of cellular changes in *O. ochengi* onchocercomata treated with different regimens of oxytetracycline [20]. Eosinophils were implicated in worm killing because a relatively short, non-adulticidal antibiotic regimen caused a transient depletion of *Wolbachia* and a partial transition in the nodule towards eosinophilia. This was reversed when the endosymbionts recrudesced, leading to restoration of the neutrophil population and survival of the adult worms. However, without a concurrent comparison with non-antibiotic chemotherapy, the possibility that the observed eosinophilia was simply a secondary response to worm morbidity could not be excluded. In the present study, we rigorously evaluated the effects of an arsenical compound, melarsomine, on *Wolbachia* to confirm that it had no direct bacteriostatic or bactericidal activity and thus could serve as a control adulticide. Data obtained from *in vitro* treatment of the Aa23 cell-line demonstrated no deleterious effects of melarsomine on the endobacteria and this was corroborated by *ex vivo* analyses of *Wolbachia* in onchocercomata, where any reduction in endosymbiont density was secondary to disruption of worm integrity.

Oxytetracycline treatment elicited a relatively early infiltration of eosinophils and extensive degranulation on the worms surface that (coupled with striking ultrastructural abnormalities at 12 wpt) are strongly indicative of immune-mediated damage. Importantly, during the initial wave of eosinophil degranulation at 8 wpt, these cells exhibited their most intense activity against morphologically normal, reproductively active worms, with lower levels of degranulation observed against parasites that already showed pathological signs. This suggests that moribund worms do not trigger eosinophil degranulation in this system, but rather that the decrease in *Wolbachia* density in viable worms is detected by the mammalian host, which responds with an intense but temporally restricted influx of DE. By 12 wpt, this response had reached an acme, with the worms exhibiting damage that was almost certainly irreversible. However, the precise sequence of events leading to the penetration of eosinophils into the pseudocoelomic cavity was not clear, as we never observed complete rupture of the cuticle in OXY-treated worms at this time-point. It is perhaps more probable that following immune-mediated alterations around apertures such as the vulva, the cells were able to diapedese internally.

Although one study has reported that doxycycline has significant anti-filarial activity against adult *Onchocerca gutturosa* worms cultured *in vitro* [27], these effects were only observed at a concentration of 100 times greater than the minimum bactericidal concentration against *Wolbachia* as determined in the Aa23 cell-line [26]; moreover, oxytetracycline had no detectable activity against *O. gutturosa in vitro* [27]. Thus, the immune system is probably required for optimal activity of antibiotics against adult *Onchocerca* spp. By contrast, we found no evidence for immunologically mediated damage following melarsomine treatment, and the very late onset eosinophilia in resolving nodules and the limited degranulation observed in this treatment group are more consistent with a role for these cells in clearance of worm fragments and in wound healing. Indeed, this process was also apparent in the latter stages following oxytetracycline chemotherapy (i.e. from 36 wpt), where eosinophil infiltration, associated with much lower levels of degranulation, continued to increase in parallel with a trend towards reduced nodule size.

Three lines of evidence support the hypothesis that the *Wolbachia*-induced neutrophilia in untreated onchocercomata protects adult worms from eosinophil degranulation. First, transcripts for bovine chemokines that are known to recruit neutrophils were significantly downregulated in the OXY group but not the MEL group, suggesting that in the absence of neutrophilic signals released directly or indirectly by *Wolbachia*, worms attract eosinophils by ‘default’. Second, at the critical time-point of 8 wpt, eosinophil degranulation was inversely correlated with neutrophil counts. Third, as discussed above, recrudescence of *Wolbachia* can reconstitute the neutrophilia and ‘rescue’ the worms during the early stages of eosinophil infiltration [20].

This manipulation of the local inflammatory response by *Wolbachia* represents a defensive mutualism that is related to, but distinct from, the protection against pathogens that it confers in arthropod hosts such as *Drosophila melanogaster* [6,8]. Indeed, in filarial worms, the host of the symbiont is itself a pathogen, and the external threat from which it requires defence is the immune system of a third organism. Closer parallels in arthropod hosts have been recently identified in two insect pests of plants. The leaf-mining moth *Phyllonorycter blancardella*, which appears to use *Wolbachia* to manipulate cytokinin levels in the leaves of apple trees, maintains ‘green islands’ in otherwise senescent leaves that facilitate the nutrition of the larval stages [28]. Furthermore, larvae of the beetle *Diabrotica virgifera virgifera*, a root pest of maize, can downregulate immune defence genes in the host plant when infected with *Wolbachia*, but loses this capacity after antibiotic treatment [29].

Since filariae have several life cycle stages and dimorphic sexes that reside in different anatomical compartments in both vertebrate and arthropod hosts, it is probable that the role of *Wolbachia* is modulated during the worm development, as has been observed in the mutualism between the entomopathogenic nematodes *Heterorhabditis* spp. and *Steinernema* spp. and their enterobacterial symbionts [30]. Haem biosynthesis can be plausibly linked to the production of ecdysteroid-like hormones that may regulate filarial moulting and embryogenesis, providing a mechanism by which the antibiotic therapy induces embryostasis and retarded larval development [15]. However, these processes fall short of adulticidal effects, and do not provide an explanation for the role (if any) of *Wolbachia* in adult male worms. The ‘patchy’ distribution of *Wolbachia* among filariae (some genera with almost all members infected; other genera with only one member infected [2]) strongly implies that *Wolbachia* may confer a relatively minor evolutionary advantage during the initial contact with a population of new hosts, such as additional nutrients during periods of resource stress [31], or an immune evasion strategy that complements the numerous mechanisms already available to the worms. The perhaps unparalleled success of *Wolbachia* as a symbiont may hinge on its ability to foster dependency by driving the loss of related functions from the nuclear genome of the host.

In this respect, the only known member of the genus *Onchocerca* that lacks *Wolbachia*, *Onchocerca flexuosa*, provides the exception that proves the rule. Partial sequencing of the nuclear genome of this species has demonstrated that it harboured an ancestral symbiont, as it contains over 100 *Wolbachia* DNA fragments [32]. Unlike *O. ochengi* and *O. volvulus*, which establish remarkably persistent infections, *O. flexuosa* has a lifespan estimated at only 1 year [33], with eosinophils constituting a prominent component [34] of the onchocercomata in its cervine host. Since *O. ochengi* and *O. volvulus* take almost a full year to develop patent infections, *O. flexuosa* appears to have adopted an alternative reproductive strategy following loss of *Wolbachia*, in which it may have accelerated its development to sexual maturity to avoid elimination by eosinophils. By contrast, the fact that adult female worms of *O. ochengi* and *O. volvulus* can reproduce continuously for a decade, while in an almost entirely sessile state in immunocompetent hosts, contends as yet another fascinating example of evolutionary innovation in *Wolbachia*.
